# Discussions before the Ohio State Dental Society, Dec. 5, 6, 7, 1872, on a Paper by Dr. D. C. Hawxhurst, of Michigan, on “The Effects of Fetid Breath upon the General System.”

**Published:** 1873-04

**Authors:** 


### Discussions before the Ohio State Dental Society, Dec. 5, 6, 7, 1872, on a Paper by Dr. D. C. Hawxhurst, of Michigan, on "The Effects of Fetid Breath upon the General System."

Dr. Rosson, being called upon, said he considered the subject treated of in the paper one of very great importance to the dental profession, but confessed that though he had given it some thought, he had not thoroughly investigated it, and could not explain intelligently what the gases of bad breath are, and how they are generated in the mouth, nor how they act upon the dentist in operating. All dentists know from experience how disagreeable it is to operate for a great many patients, that appear when they come to the office to be cleanly, and yet whose breath emits an odor or effluvia that is extremely disagreeable. He had no doubt the injury received from that source was very great. He related an instance in which a distinguished professor had become so well convinced, that the gas, arising from the ignition of matches, was so injurious to him, resulting in breaking down his health, that he had matches manufactured for his own use free from sulphur. It was well known what a terrible thing blood poison was, and how hard to get rid of, and, as he understood it, these gases which act upon the system are carried through it by the action of the blood. He was glad the subject had been brought before them. He would go home and investigate this matter, as he did all the subjects discussed in their meetings, and learn all he could about it, and apply it to himself. If every member would pursue this course, he thought great benefit would be received from their discussions.

Dr. Harroun said he was greatly interested in the paper, and much pleased to know that it was to be published. A great deal of truth he believed had been brought to light in it. He thought it should set them to thinking how they might prevent the injurious effects of these gases, and prolong life by the proper care of themselves and their patients in this respect.

Dr. J. B. Beauman thought there was an important Scriptural quotation they might apply, "How shall we see clearly to pluck the mote of our brother's eye when there is a beam in our own eye." He had met dentists whose breath was as offensive to him as that of his patients. In looking at this question, he thought it would be well for them to sweep their own houses before complaining of their neighbor's houses. It behooved every dentist to look well to his own mouth before he says anything about the mouth of his patients. He feared most of them were careless in regard to the cleanliness of their mouths.

Dr. W. M. Herriott thought many dentists are too careless in regard to the condition and size of their operating rooms. Many throughout our State and the United States take great pains in fitting up their drawing room, so that it shall be a cheerful and inviting place, while they coop themselves up in a little room, some not more than eight by ten feet. That is their living room filled with foul breath, and then they re-breathe the matter thrown off from their own systems and the systems of their patients. It is surprising that under such circumstances they have as good health as they do. Good light and good air are indispensable to the dentist in his operating room. His operating room was made the best room he had, being large, well ventilated, and furnished with good light.

Dr. C. M. Kelsey regarded the paper, read by the gentlemen from Michigan, as quite a valuable addition to dental literature. He considered it one of the best papers ever read before this society.

Dr. J. M. Porter thought there was one thing that many dentists were in the habit of doing when operating, that produced injurious results. That was opening the lips and keeping the mouth open while operating. This was to be considered an unfortunate habit anywhere, in doors or out. It was unnatural, and must produce abnormal results. Especially was it injurious when standing over a patient. If a man was fortunate in possessing a heavy moustache, and kept his mouth shut, he would prevent much of the evil effects that might otherwise result. If the mouth is kept open the operator must necessarily inhale everything that comes in contact with it. Nature has provided some means to shut off deleterious gases—through the nostrils is the natural way, and through the mouth the unnatural way.

Dr. J. Taft said there was more that might be said in that direction. The mouth when open not only inhales foreign substances, or materials that ought not to go to the lungs, and which would not go, to the same extent, at any rate, through the nostrils. The decomposition of foreign substances in the mouth is a very prolific source of obnoxious gases, and that decomposition is far more rapidly accomplished when the mouth is open. The mouth should always be kept closed, except for the reception of what goes into it. When the mouth is open there is a vitiation of its fluids injurious to the teeth and general system. The elaboration of agents that operate to produce decay of the teeth, is far more liable to be accomplished when the mouth is open, and thus the teeth suffer in consequence. Those persons—other things being equal—who are in the habit of keeping the mouth open by day or night, will have the more rapid decay of the teeth These are practical details that should claim the attention of every dentist, especially so far as his directions to mothers and children are concerned; we should give mothers a right view of these things, and use our influence to have their children contract correct habits in this respect. Very much would be attained in this way for the welfare of children in regard to their health, and the preservation of their teeth.

It is an interesting subject to consider this matter of the ability of the lungs, through their membraneous tissue to receive into and through them the agents that come in contact with them. My impression is that the paper presented last night put that matter pretty strong; not too strong, perhaps, but there are modifying circumstances, and we are not to infer or come to the conclusion that whatever comes in contact with the lungs will be greedily taken into them. My impression is that gases are constantly passing in both directions, in-coming and out-going. We do not take in these gases in a concentrated form, but they are largely diluted by the atmospheric nitrogen and oxygen taken in with them.

In one respect what the paper said is true, that is the facility with which the lungs will take into them, and transmit to the blood, foreign substances. This is very clearly set forth in the paper. But let us not conclude that everything that comes in contact with the lungs, either in the form of gases or fluids, will be taken into them. They have some ability to make a selection, not however to exclude all injurious agents, but they certainly will exclude some agents. There are some fluids which if inhaled, will make manifest impressions, and will not be received. Take, for instance, nitric oxide gas, which if taken into the lungs will produce spasms and a closing up of the lungs, so that they will not receive it. He did not know that a different idea was entertained by the writer of the paper, but some might have received such an idea.

In conclusion he remarked that the subject was a very interesting one indeed, and should be more thoroughly understood by all members of the profession. It is a practical matter not only for the dentist, but for his patients. In all such things the knowledge of the dentist, however great it may be, can be, and should be exercised by him not only for his own benefit and protection, but for the benefit and protection of the patients committed to his care.

Dr. H. A. Smith regarded Dr. Taft's remarks as very pertinent to the subject. He desired, in speaking briefly upon the subject, to refer to one or two points brought forward by Dr. Porter. With regard to this matter of inhaling injurious gases during operations, he had thought sometimes some kind of a mechanical respirator might be invented, so that while breathing the air it might be strained. Dr. Taft had suggested that if the mouth were kept closed it would prevent the taking in of deleterious gases. Where he had a patient with an offensive breath he had been in the habit of pulling his moustache down over his mouth, and keeping the mouth closed in order to prevent the inhalation, or strain out the offensive materials, that are always more or less given off. Persons working amid the dust of metals wear a cotton web over the mouth to prevent the dust from being received into the lungs.

He did not concur with Dr. Taft in the opinion that the lungs have the power of selecting what is taken into them, except that deleterious substances sometimes, when taken in, will produce irritation.

He did not think the writer of the paper stated too strongly the cause of odors as from diseased teeth, etc; but we often find patients who have fetid breath which can not be traced to anything, or its source detected. The causes in some cases are very obscure, and one which he thought physicians knew but little about. He knew but little about this himself, but had thought much about it.

Carbolic acid administered in small doses would correct this matter to some extent. His attention had been called to that by a physician of Cincinnati. Dentists could, of course, distinguish whether this fetid breath proceeded from the teeth or respiratory organs.

He was glad this subject had been taken up, and treated so well by Dr. Hawxhurst. The paper evinced considerable research and labor. We needed more literature on the subject.

Dr. Hawxhurst said that a knowledge of this subject is likely to be very useful to the practitioner. The question, how to keep the air which passes to the lungs pure and wholesome, is one of vital importance. Out of doors the air is generally pure; and yet in going along the streets we often come in contact with foul air, and unpleasant odors; but we have little control over the out-door air. How to keep the air of our rooms from being impure and disease-producing is a more important and practical inquiry What gases are liable to vitiate the air of our rooms? Our carpets may become saturated with gas that will be driven off by heating the rooms, and will be injurious; the varnish on the furniture gives off gas, and wall-paper, when the room is warmed, gives off gas. He had seen an account of two instances last winter, in which disease had been produced by the gas arising from drugs used in coloring the paper of a room.

Again there are gases arising from the drugs used in dental offices, from the cloths that cover stands, and from the upholstering of chairs and sofas, from the drawers of cabinet cases, and the meshes of all bodies loose in texture; a great deal of deleterious gas is discharged when there is an unusually warm fire, heating up the contents of the room; so that the sources of impure and injurious gases are very numerous. Sometimes the skin itself gives off gas; sometimes the hands of the dentist throw out such an unpleasant odor as to be annoying to the patients.

From the lungs come some unpleasant odors, and volatile principles, that it is not always easy to account for. He had been told that their origin is very obscure, and that it is useless to spend time in research on this point. True, the nature and sources of these seem to be hidden, but he thought they might be ferreted out. The subject was one of very great interest, and requires much investigation. If we understood the physiological mutations of matter in the system, as we might, and the laws of experimental logic, we would be able by experimental tests to discover the causes of bad breath as exhaled from the lungs. Except in cases of local disease of the organs themselves, those fetid odors that come from the lungs as a part of the expired air, must have made their way through the lung tissues directly from the blood. How do they get into the blood? The matter from which they arise is ingested with the food, or as a part of it, or it may be results in part from the degeneration of alimentary substances during an ill-performed digestion; foreign and injurious compounds may be thus developed in the body—compounds which, going the rounds of the circulation, will volatize in the lungs, and spoil the breath Or, the vitiating odor may be derived from destructive assimilation, and may represent the effete and worn-out atoms of the system, the waste matter and debris of the animal organism, which have not been eliminated from the system by a sufficient activity of the excretory organs.

Whatever we eat goes through the digestive process perfectly or imperfectly, and finally passes into the blood, and arrives at the lungs. So, if you eat turnips or celery, the blood very soon contains the essential oils of these vegetables. If we eat onions we find the blood, perhaps, in half an hour, has given the odor of this food into the lungs and into the breath. Alcohol will thus make its way to the lungs in about three minutes; it simply takes this route of exit from the body, and on the way spoils the breath. Almost all the essential oils which are taken into the stomach as a part of food are thrown off from the blood in a volatile state, and pass through the lungs into the breath.

In general, deleterious gases will be thrown out from the lungs whenever one or more of the excretory organs is inactive. So if the kidneys are not sufficiently active, matter which should be pumped off by them may pass off through all the other emunctories, but, so far as they are volatile, through the lungs. In cases of inactivity of the colon there may be left volatile animal matter in the blood, which ought to have been drawn off through that passage. If so, it will be eliminated in some direction, and very likely through the air passages. In crowded, ill-ventilated habitations, more or less, morbid matter may be taken up by the absorbents of the skin, thrown upon the blood, and afterwards, perhaps, carried to the lungs for elimination.

Another matter that is full of interest in connection with this subject relates to bad breath from suppressed menses. The menstrual fluids contain many volatile matters that saturate the blood when retarded or suppressed, and find their way out through the lungs. He had observed frequently a very peculiar and unpleasant odor in the breath of women, and had discovered in the case of two girls, who exhibited the same qualities of breath, that the menstrual flow was entirely suppressed. On talking with Dr. Robbins, of Meadville, he had obtained other instances of the same kind. During his investigations of this subject, he had observed so many instances in which the suppression of the menstrual discharge was accompanied by this peculiar fetor of the breath, that he had lately set it down as one of the causes of bad breath. It seems that this matter, which should be drawn off from the blood monthly by way of the uterus, if retained in the system, may be vicariously discharged through the pulmonary surfaces.

Dr. Rehwinkle asked Dr. Hawxhurst, if he had ever observed any particular conditions of the breath accompanying symptoms of insanity.

Dr. Hawxhurst replied that he had not thought much about that. Wherever insanity occurs there is generally some obstruction of the physical functions. We all of course recognize that the mind is dependent upon the body, and that mental action is modified by physical conditions. May it not be that the different kinds of tissue-changes, that go forward during insanity, bring about a different composition of the blood, and that thus unusual and abnormal compounds or substances are presented for elimination at the lungs.

In general, when physiological action is normal, there is nothing in the system that will produce bad breath. The carbon and hydrogen when oxidised are inodorous. The essential oils, and all impurities, when sufficiently burned down by oxygen, are inodorous. But when these are excessive in quantity or peculiar in nature, as they might be in insanity, this oxidation may not be complete.

Dr. Rehwinkle remarked that though he was entirely unable to throw any light upon the subject, yet it was a fact, that in marked cases of insanity, bad breath was perhaps one of the most common and peculiar features. It had always been an interesting subject to him, but he could never satisfy his mind why this should be. He had never been able to decide that it is in consequence of the dependence of the mind upon the physical structure, because in many instances the functions would all seem to perform their work with great regularity; the digestive organs, etc., all in excellent condition, so that it seemed there was nothing in the physical condition of the person concerned, to justify you in supposing that the fetor of the breath arose from any cause in operation here.

In regard to bad breath in females, occurring in consequence of the suppression of the menses, he had made no observations. It was entirely a new idea to him, and had never presented itself to his mind in this shape before. It was however, he thought, a matter full of interest.

Dr. H. A. Smith had observed that at that period in females, termed the change in life, when the menses are suppressed by the order of nature, their breath is especially offensive. He had had opportunities to observe this in several instances. After this period had passed, and the system had conformed to nature's demand, this offensive breath passed away.

